# Current Scenario of the Tehran Municipal Solid Waste Handling Rules towards Green Technology

**DOI:** 10.3390/ijerph16060979

**Published:** 2019-03-19

**Authors:** Parveen Fatemeh Rupani, Reza Maleki Delarestaghi, Hossein Asadi, Shahabaldin Rezania, Junboum Park, Madjid Abbaspour, Weilan Shao

**Affiliations:** 1 Biofuel Institute, School of Environment and safety Engineering, Jiangsu University, Zhenjiang 212013, Jiangsu, China; parveenrupani@gmail.com; 2 Department of Environmental Engineering, University of Tehran, Tehran 141556619, Iran; Reza_maleki@ut.ac.ir; 3 Chemical Engineering, University of Pyam Nur, Tehran 1659639884, Iran; asadi.hossein90@gmail.com; 4 Department of Civil and Environmental Engineering, Seoul National University, Seoul 08826, Korea; Shahab_rezania89@yahoo.com (S.R.); Junbpark@snu.ac.kr (J.P.); 5 School of Mechanical Engineering, Sharif University of Technology, Tehran 11155-9567, Iran

**Keywords:** urban waste, municipal solid wastes, compost, Kahrizak-Tehran

## Abstract

This study aims to study the waste management process and recycling of municipal waste in Tehran. Currently, Kahrizak is the defined landfill area which collects the waste generated from 22 districts of Tehran. The organic wastes undergo to the windrow composting method in order to manage the partial of the waste generated in the city. Samples from the compost pile generated in Kahrizak were examined to evaluate its fertilizer value to be used further by the farmers. The results show that the obtained compost does not reach the acceptable quality to be used further in agriculture, due to lack of homogeneity, aeration and presence of heavy metals. Overall, it has been concluded that, due to the improper waste segregation and management prior to sending to landfill and presence of non-organic materials such as hazardous metals and medical wastes, causes difficulties in proper waste management, implementation and producing high quality compost. Based on this study the existence of stakeholders, society, economy and proper handling rules can effectively improve the waste management system in the country and leads to the sustainable green environment.

## 1. Introduction

The rapid population growth and industrialization led to increased generation of solid wastes, which consequently has become one of the biggest global challenges [1]. The major part of the urban wastes generated in developing countries are consisted of organic wastes such as food, paper, weeds, garden wastes and so forth. In Iran (Islamic republic), with having 81 million population as of 2017 [2], majority of the waste generated in urban areas were consisted of 70% organic waste [3]. Previous studies show that the organic waste constitution in Europe and United States were accounted only 40% and 58% accordingly [4]. Disposing of the solid wastes on open lands or in the improperly designed landfills (e.g., in low-lying areas) can cause adverse effect to the environment as well as human health. Re-using of organic wastes differentiated from the municipal waste for agricultural purposes is among the stablished rules worldwide. Among different approaches of mitigating the waste, composting is one of the suitable biological methods to transform the organic wastes into useful soil amendments [5,6,7]. Composting is a process in which organic waste is degraded by microorganisms under aerobic conditions [8]. During this process the available nutrients in the waste converts into the components available for the plants through microbial action. The process is under thermophilic phase (45 to 65 °C) which can effectively reduce the mixture volume by 40–50% [9]. Although composting is not considered among the new applied technology but till date it is one of the best solid waste management strategies to replace the landfilling technique [10,11]. During the composting, toxicity level of the components reduces and the final product (compost) will resulted in improved soil quality [12]. In addition, according to Lou and Nasir [13], composting process can reduce the production of greenhouse emission.

Mir and Nabavi [14], reported that about 70% of the waste produced in Iran is considered wet waste and 30% is dry waste. Authors have added that usually 83.6% of the wastes produced in Iran ends up in landfill, while 10.5% enters the compost process and only about 5.9% is recycled. However, due to the poor waste management in the country hazardous household wastes such as paint, batteries and poisonous utensils and hospital wastes are not collected separately, which leads to environmental health problems [15,16,17]. 

**Figure 1:**
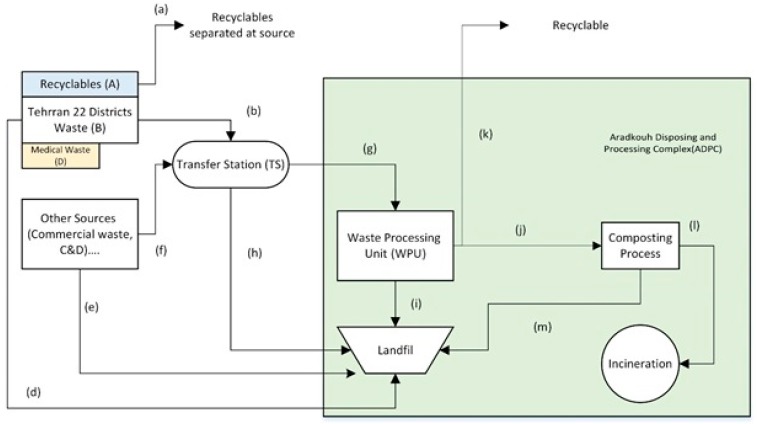
Waste management flow in Tehran (letters (a)–(k); Adopted from Malmir et al. [20].

The Tehran Waste Management Organization (TWMO) is one the responsible organization for the waste management strategies in Iran. Tehran as the capital city of Iran had two main landfill sites named as Abali and Kahrizak. Each site had the capacity to receive almost half of the MSW generated from the city which was approximately 2.6 million tonnes in a year. However, due to the open dumping and the unsanitary disposal of the wastes, which resulted in ground water contamination and sever health problems, the site closed in 1991 [18]. Form then after, Kahrizak Landfill is the main site for MSW generated from the city and renamed as “AradKouh landfill” [19]. Figure 1 shows the current waste flow status in Tehran, adopted from Malmir and Tojo [20]. According to the statistics of the TWMO there are four sources of waste and recyclables: (A) recyclables generated in the 22 districts of Tehran city; (B) MSW generated in these districts; (C) MSW generated from other sources (companies and rural areas); and (D) medical wastes. Overall, sum of the waste generated in (A) and (B) can be regarded as the ‘exact waste’ generated in 22 districts of Tehran.

There are three main pathways of waste generated from 22 districts of Tehran city, as shown in Figure 1. The first is the recyclables segregation at source point (a). The recyclable waste materials contained stale bread, plastics, paper and card-board, metal, glass and so forth. The second pathway of MSW from districts is a routine collection (b) performed by the TWMO. A daily door to door waste collection service is provided every night. Collection vehicles (mechanized compacting vehicles) collect the waste and transport it to the defined 11 transfer stations (TSs). Hence, the main duty of the TS is just to transfer MSW from the collection vehicles to semi-trailers in order to transport waste economically and efficiently, since the Arad Kouh Disposing and Processing Complex (ADPC) is located about 40 km away from the city centre. The third pathway is the direct disposal to the landfill (c). The types of waste that are directly disposed of into the landfill include sludge, soil and yard trimmings, generated mainly from the public works site. 

Medical waste will directly transport to the ADPC (d) and is disposed of in a sanitary mode at a designated location in the landfill. However, little attention is given for this part due to the unawareness and lack of knowledge. Other MSW generated in commercial and rural areas (C) are also treated by the TWMO. In this case part of rural waste is transported to the 11 TSs (f) and the rest is sent directly to the landfill in the ADPC (e).

Most of the waste delivered to TSs is taken to the waste processing unit (WPU) located in the ADPC (g), however, part of it sent from the TSs directly to the landfill in the ADPC (h). The amount of wastes (g) transfers to the WPU is directly proportional to WPU capacity. At the waste processing unit in the ADPC, the waste is separated into recyclable materials (k), a fine fraction composed of mainly biodegradable organic matter (j) and a coarse fraction (i) (called reject materials) which is mainly plastic and paper that are not recyclable because of contamination. Operation of the WPU is assigned to private contractors by the TWMO and recyclable materials (k) separated by these contractors are sold to recycling facilities, which are operated by both private companies and public institution. Materials separated as recyclables are cardboard, plastic, polyethylene terephthalate (PET), aluminium and so forth. The fine fraction is sent to the composting facility located adjacent to the WPU and turned into compost. There are four inflows to the landfill: direct disposal from districts (c), direct disposal from other sources (e), direct disposal via TSs (h) and reject materials from the WPU in the ADPC (i).

The factors affecting the physical and chemical characteristics of the compost product can be divided into two general sets of the mechanical conditions and environmental conditions. Mechanical conditions are consisted of input materials to the system, process conditions, process time. Etc [21]. The most important environmental conditions are the amount of food, the proportion and the balance between available food, aeration, the ratio of carbon to nitrogen, temperature, pH and particle size [22,23]. Therefore, in this paper, the quality of the compost produced from the composting site was evaluated to investigate its final characteristics to be suggested as a good compost to be used by the farmers. 

## 2. Materials and Methods

### 2.1. Description of the Study Area

Iran has an area of 1,648,195 km^2^ (636,372 sq. mi). It lies between latitudes 24° and 40°N and longitudes 44° and 64°E. Tehran, the capital city of Iran, is located at the northern part between 35.68° N and 51.38° E latitude. The city is divided into 22 municipal districts. Aradkouh, a compost plant with an intake capacity of 500 t day^−1^, began operating in 1998 in the vicinity of the AradKouh Landfill, located at 40 km away from the city centre. The altitude above sea level of this region varies from 1020 to 1060 m, the average annual temperature is between −5 and 40 degrees Celsius and the average rainfall and evaporation are 240 and 250 mm, respectively.

**Figure 2:**
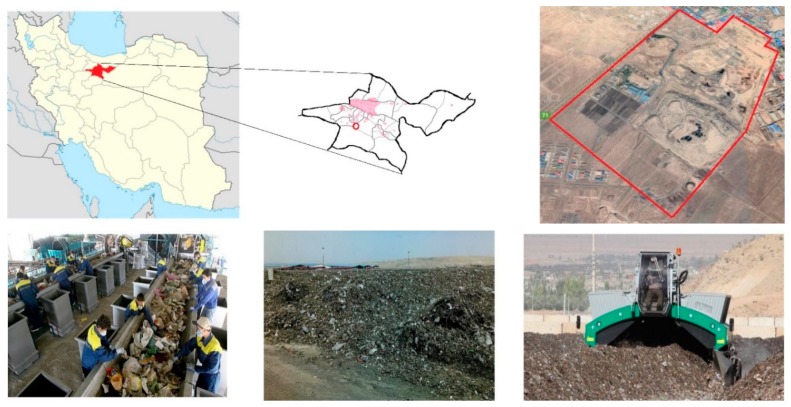
Location of compost site in Aradkouh processing and disposal complex.

Aradkouh Landfill has been divided into 4 parts as shown in Figure 2. These 4 sections include processing lines, composting plants, landfills and waste incinerators. A majority of the waste produced in the city of Tehran will dump at the Processing and Disposal Centre of Aradkouh. From those the recoverable materials are separated, part of the waste from the processing system enters to the Landfill zone and the rest enters the bioprocess (compost) plant. In recent years, a waste incinerator with a capacity of 200 tons per day has been built in the area [24]. However, small portion of the wastes have been imported daily to the incinerator. Figure 2 shows the Aradkouh location and its waste processing disposal complex units. The composting process at the centre is carried out using the windrow method, the width of the masses is about 2–5 m and the of the compost pile could reach between 1 and 2 m height. 

### 2.2. Physico-Chemical Properties of Compost

This research was carried out at the processing and disposal centre of Aradkouh, where the composting piles were located. The compost piles were composed of urban wastes generated in 22 districts of Tehran, after segregation of the recyclable materials. The approximate length of the piles was about 50 m with a width and height of 1.5 and 1.2 m respectively. In this research, the physical and chemical properties of compost mass were investigated over a 70 day period. The piles were aerated manually every 10 to 15 days, while no additional moisture was added to the waste mass during the process.

At 7 day intervals, around 5 kg samples of compost were collected at depths of 120–150 cm from each windrow heap. The samples were oven dried (100 °C), ground and stored in labelled plastic bags for further analysis using recommended standard methods. The temperature of the compost was monitored by thermometer, following the APHA [25] method. The pH of the compost was determined by using a doubled distilled water suspension in the ratio of 1:10 (w/v) using pH Meter. The amount of total organic carbon (TOC) and total nitrogen (N) were measured with a CHNS analyser (Elementar Vario MARCO cube CHNS, Frankfort, Germany), while the total amount of potassium (K) and heavy metals (Pb, Ca, Hg, Cr) were determined after digesting the sample in a tri-acids mixture (HNO_3_ + H2SO_4_ + HClO_4_) by flame photometer [26].

## 3. Results and Discussion

**Figure 3:**
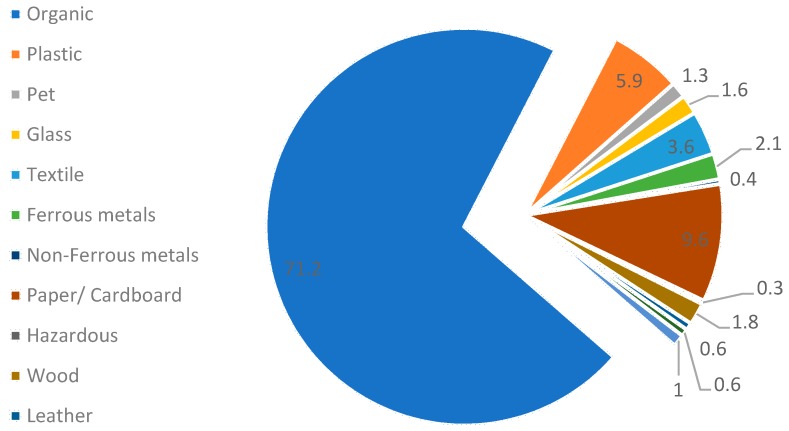
Waste Composition breakdown at Aradkouh-Tehran.

Figure 3 shows the detailed composition of Tehran’s waste. Based on the results, 71.2% of the waste collected from 22 municipalities consisted of organic wastes. The high proportion of organic waste composition creates high potential for using compost processes to utilize it. Among other recyclable wastes, paper and plastics account for 9.6% and 5.9%, respectively, comprising the biggest portions of the waste piles. Hazardous waste and leather accounts for the lowest portion of waste, which is due to the mismanaged strategy of collecting such wastes at the initial point. However, the amount of waste varies seasonally. 

### 3.1. Moisture Content

**Figure 4:**
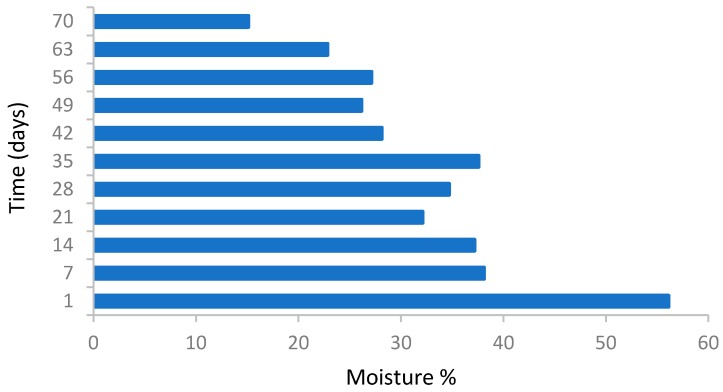
Moisture variations during the composting process.

Figure 4 shows the % moisture content of the mass over 70 days of composting. The initial moisture level was 58% which decreases significantly to 15%. The reduction in the moisture level indicates the degradation of the organic matter in the mixtures. Matrix structure and water content change dynamically during the composting process. The decomposition process reduces particle size and increases matrix dry bulk density, leading to a reduction in total porosity [7]. The heat and air-flow generated during composting evaporate significantly more water than is produced and tend to dry the material out [27]. Maintaining the moisture content of the waste mass (40–55%) is vital for the survival of microorganisms, as it is necessary for the metabolism as well as reproduction during the composting process. On the other hand, the needed food for the use of microorganisms is transmitted through water [23,28]. Therefore, the presence of sufficient moisture in the waste mass during composting process is of particular importance. Based on O’Leary [29] and Iqbal Muhammad [30], the optimum moisture for biological process in controlled conditions can be up to 60%, however in processes with less control, the optimum moisture content was about 50% to 55%. An efficient range in waste mass is important because if the moisture content is less than 40%, the growth of microorganisms greatly reduces and thus the biological process slows down [7]. Moreover, the authors stated that when the moisture content of the mass is at a high level (for example, more than 65 Up to 70%), it is likely that the pore space between the mass particles become filled with water and the movement of oxygen through the pores gets disrupted. This can prevent the effective movement of air and result in the generation of anaerobic conditions and an unpleasant smell [23,30]. On the other hand, excessive moisture could lead to the production of leachate in the waste mass during the compost process [31]. 

According to the obtained results, the moisture content of the waste masses was 40% from the 5th day of composting onwards, suggesting a low moisture content of the wastes during the process. A low moisture level in the heap can cause disruption to the composting process and disturb efficient degradation. Effective factors in decreasing the amount of moisture during the process include the activity of microorganisms, the high evaporation rate in the compost without the addition of extra water during the composting process.

### 3.2. Temperature

**Figure 5:**
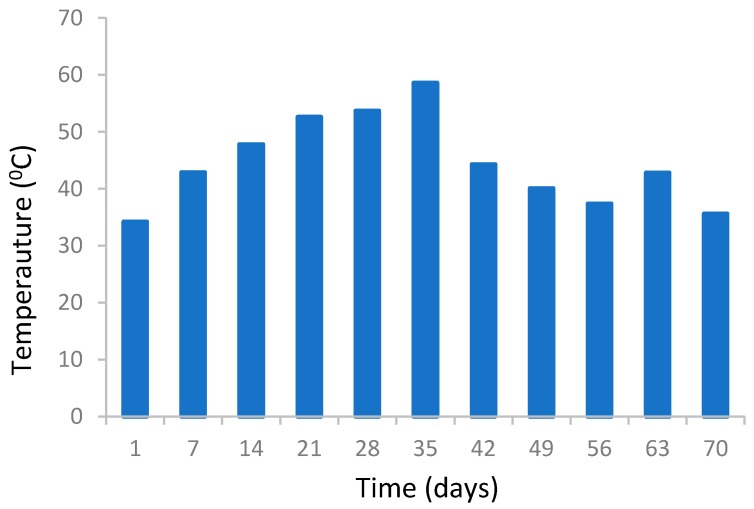
Temperature variations during the composting process in waste mass.

Temperature is one of the most important factors in the development of biological processes [28]. Therefore, keeping the temperature in the optimal range can help the process of organic material degradation by microorganisms, as well as the time reduction of the process. There is a direct correlation between the temperature inside the mass and the activity level of the microorganisms. Temperature increase happens as a result of biological activities [30]. According to Mason and Milke [32], a temperature between 50 and 65 degrees Celsius is the optimum temperature for the activity of microorganisms and an excessive increase in temperature (65–70 °C) reduces the population of microorganisms. However, increasing temperature in waste mass causes the destruction of pathogens as well as weed seeds [32,33]. Aeration is generally used to control the temperature. As it has shown in Figure 5, temperature changes are depicted during the compost process. According to the data obtained, temperature variations during the composting process were between 35 to 45 °C. The maximum temperature achieved at the 35^th^ day of the composting process was up to 60 °C. The increase in temperature between days 20 and 30 indicates the thermophilic phase in which micro-organisms attack the soluble, degradable compounds [34]. On the other hand, low temperature, observed from day 40 onwards, helps to conserve N in composted materials, since a high temperature can cause high losses of N in the form of NH_3_ during the early stage of the composting process [35]. 

### 3.3. pH

**Figure 6:**
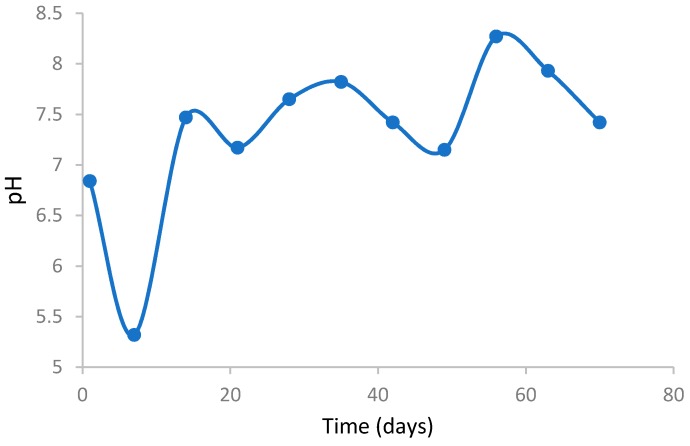
pH variations during the composing process in waste mass.

Figure 6 shows the pH variation of the compost heap during the composting process. During the first 5 days of the composting process pH shows acidic stage ranges between 5–5.5. This stage indicates the initial composting process which verifies significant stabilization of ions. This is in parallel to results shown in Figure 6, where the moisture content also reduces significantly. According to Petric and Selimbašić [36], increased activity of microorganisms in the mixture will lead to production of CO_2_ in the mixture and the combination of this with the moisture level of the mass results in the generation of carbonic acid which increases the acidity level of the mixture. Sundberg et al. [9] reported similar results during the composting of household waste. According to the data obtained in this study, the pH level neutralizes after 15 days, ranging between 7–7.5. This indicates the gradual stabilization of the mixture.

### 3.4. C:N Ratio

**Figure 7:**
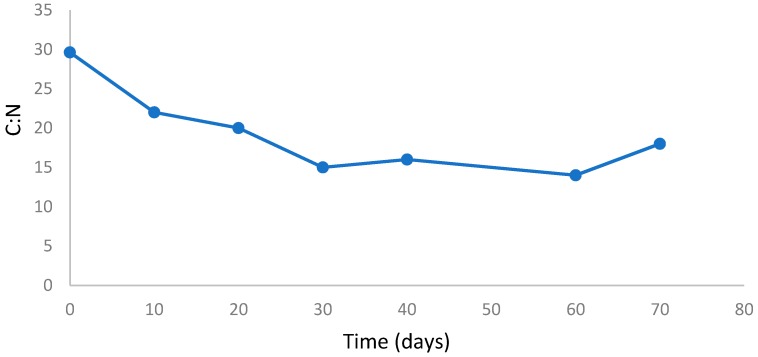
C:N variations during the composing process in waste mass.

C:N is an important parameter in defining the maturity of the compost, which depends on the type of organic material. Figure 7 shows the C:N ratio of the composting pile over 70 days. As illustrated in Figure 7 the C:N ratio decreased below 30 from 20 day onwards. According to Morais and Queda [37], a C:N ratio less than 15 is more preferable for the agronomic use of compost. In this experiment a C:N ratio of 15 was found at day 30, which shows the compost mixture had stabilized. Zmora-Nahum et al [38] found that nitrogen is the main factor in the composting process. They have reported that a high C:N ratio is due to nitrogen deficiency in the mixture. Different waste composition may result in a different starting C:N ratio, which could affect the C:N ratio of the final product. For instance, if the mixture contains more carbon source material, it results in nitrogen deficiency and vice versa, the nitrogen-based waste may increase the ammonia concentration of the compost which could result in generating more NO_2_ gas in the system. Therefore, in order to achieve a stabilized mixture, the input material has to be taken into account prior to the composting process. 

### 3.5. Heavy Metals

According to the existing standards, the amount of lead in the waste mass is too high in Iran but the remaining heavy metals are less than the permissible value. One of the main factors of the excessive presence of this heavy metal is the existence of materials like batteries in waste mass. Based on the results obtained, the concentration of lead (pb), is higher than the threshold standard of the country (Table 1). The excess heavy metal concentration is due to the presence of batteries, electronics, cosmetics and so forth. High concentrations of heavy metals can limit the exploitation of the waste for the composting process [39,40]. 

The most common metal components are cadmium, chromium, nickel, zinc and lead which varies depending on the amount of heavy metal materials in the waste [41,42]. These components can remain in the soil for the long term, therefore, use of contaminated municipal waste compost may increase the mobility of heavy metals in the soil and increase the absorption of these metals by plants [43]. Hence, by entering into the food chain this results in the human health and environmental safety problems [39]. Moreover, the use of low-quality composts in the soil results in the accumulation of heavy metals and nutrients that destroy soil structure and ultimately leak into underground water [44]. On the other hand, these materials have adverse effects on the health of humans and animals, because heavy elements enter the soil, underground water and then enter the food chain [45]. Table 1 shows the amounts of heavy metals in the studied mass.

## 4. Conclusions

The current research contributes to waste management strategies, focusing on the Kahrizak landfill, Tehran. The study indicated that the different types of wastes such as the wastes produced from the 22 districts of Tehran city, along with the hospital waste and hazardous waste have been sent to the landfill. Currently there are not proper waste management and handling rules, applied in Tehran which leads to several problems. Moreover, the production of different organic and inorganic waste in the city which includes hazardous waste (dye, batteries, etc.) and hospital waste causes difficulties in proper management and compost production. 

According to the data obtained, the reduction of the moisture content in the compost leads to a decrease in the quality of the final compost obtained. In order to provide optimum moisture content in the pile, adding extra water by reusing the output leachate produced from the disposal site is suggested. Although the CN ratio of the compost pile is within the acceptable range (15–20) the total quality of the compost is not very much welcomed by the farmers. This is due to the presence of heavy metals as well as undegradable materials such as glass and plastics. Unfortunately, at present, Tehran landfill is not equipped with a gas extraction system, therefore, significant amounts of methane gas enters the atmosphere. However, an organized composting process could reduce methane production during the composting process leading to a decrease in air pollution near the landfill. 

Understanding the different characteristics of the waste and its handling rules can help the city with proper waste management. In the present scenario, the construction of separate lines at the processing and disposal centre of Aradkouh, in which the segregation of organic and recyclable products can take place, will help create more qualified end products. A relationship between stakeholders, government and socially involved people will effectively help in moving the waste management of the country towards a sustainable green society. Increasing public awareness about separating waste in each household with help to improve the waste management process by providing proper collection bins appropriately designed in metallic with closed lids will also be helpful. The municipal authorities should also maintain storage facilities with maximum hygrine conditions. This process will help with the maximum segregation of recyclable material and efficient transport to the composting section, in order to produce a high quality end product.

## Figures and Tables

**Table 1:** Heavy metals concentration during composting.

Day	Pb (ppm)	Cr (ppm)	Hg (ppm)	Cd (ppm)
0	315	81.7	0.23	2.1
7	267	72.9	0.22	2.2
14	273	75.3	0.19	1.9
21	269	86.8	0.24	1.7
28	282	72.9	0.28	1.8
35	302	84.3	0.17	1.9
42	296	86.7	0.19	2
49	287	91.3	0.26	1.9
56	291	78.4	0.25	2
63	315	79.6	0.32	1.6
70	314	74.3	0.25	2.1
Final Compost	415	88.9	0.38	2
Institute of Standards and Industrial Research of Iran	200	250	5	10
